# Coronavirus Disease 2019 (COVID-19) Can Masquerade as Acute Postoperative Periprosthetic Joint Infection

**DOI:** 10.7759/cureus.7857

**Published:** 2020-04-27

**Authors:** Eustathios Kenanidis, Panagiotis Kakoulidis, Panagiotis Anagnostis, Anastasios Beletsiotis, Eleftherios Tsiridis

**Affiliations:** 1 Academic Orthopaedic Department, Papageorgiou General Hospital, Thessaloniki, GRC; 2 Center of Orthopaedic and Regenerative Medicine - Center of Interdisciplinary Research and Innovation, Aristotle University Medical School, Thessaloniki, GRC; 3 Academic Orthopaedic Department, Papageorgiou General Hospital, Aristotle University Medical School, Thessaloniki, GRC; 4 Orthopaedic Department, Papageorgiou General Hospital, Aristotle University Medical School, Thessaloniki, GRC

**Keywords:** coronavirus disease, covid-19, sars-cov-2, infection, postoperative joint infection, total knee arthroplasty, tka, pneumonia, chest infection, virus

## Abstract

Fever etiology during the first postoperative days following total knee arthroplasty (TKA) may be challenging to solve. Early periprosthetic joint infection is the main reason; however, other equally important causes must be excluded such as thrombosis, deep venous thrombosis, and chest or urinary tract infections. We report the case of a 70-year-old Caucasian female patient presented with high fever reaching 39°C, fatigue, and myalgia lasting for a week after a fully cemented primary TKA. Symptoms were falsely attributed to the surgical procedure, leading to erroneous early management and a complicated postoperative course. In the era of the severe acute respiratory syndrome coronavirus 2 (SARs-CoV-2) pandemic, a high index of suspicion for coronavirus disease 2019 (COVID-19) symptoms and viral chest infection must be raised, primarily in vulnerable patients.

## Introduction

Fever during the first postoperative days following total joint arthroplasty is not uncommon [1]. It is usually attributed locally to surgical trauma or postoperative hematoma absorption due to inflammatory cytokine accumulation [1]. The duration and magnitude of fever, as well as concomitant symptoms, may raise the concern of early periprosthetic joint infection [1]. Other, however, equally important causes must be excluded such as chest or urinary tract bacterial infection, deep venous thrombosis, pulmonary embolic events, and, more rarely, concomitant respiratory viral infections [2]. Several clinical symptoms or laboratory tests may help reach a diagnosis; however, the diversity of differential aetiologies may delay proper treatment [2-3].

We report the case of a 70-year-woman who underwent total knee arthroplasty (TKA) a week prior and presented with high fever, myalgia, and malaise. Fever was falsely attributed to wound inflammation and hematoma absorption, leading to erroneous early management and a complicated postoperative course.

## Case presentation

A 70-year-old Caucasian female patient suffering from left knee primary osteoarthritis underwent fully cemented TKA under regional anesthesia. Past medical history included arterial hypertension, long-standing, well-controlled, insulin-dependent diabetes, glaucoma, and mild obesity. The procedure was uneventful, a standard postoperative protocol was followed with partial weight-bearing, and she was discharged home 48 hours postoperatively. The patient had a low-grade fever during the first two postoperative days. Analgesics, non-steroidal anti-inflammatory drugs (NSAIDs), and anticoagulation were prescribed, as well as physiotherapy. The day after discharge, she felt unwell with muscle aching body fatigue and developed a temperature of 38°C. The clinical examination by a physician at home was focused on the surgical wound and the local hematoma absorption, and she was treated with oral antibiotics (cefuroxime 500 mg twice a day) NSAIDs, paracetamol, hydration, and observation. Attention was not turned to chest symptoms that were mild at the time with occasional coughing and intermittent chest pain. Despite antibiotic treatment, the patient had persistent periodic high fever up to 38.5°C, myalgia, fatigue, and malaise along with mild coughing. On the sixth postoperative day, a second physician consultation by the telephone this time switched the antibiotic therapy to moxifloxacin 400 mg twice a day. The high temperature did not subside, the antibiotics made no difference to the symptoms, the wound remained red and mildly inflamed, coughing became intense, but the patient did not have difficulty in breathing.

**Figure 1:**
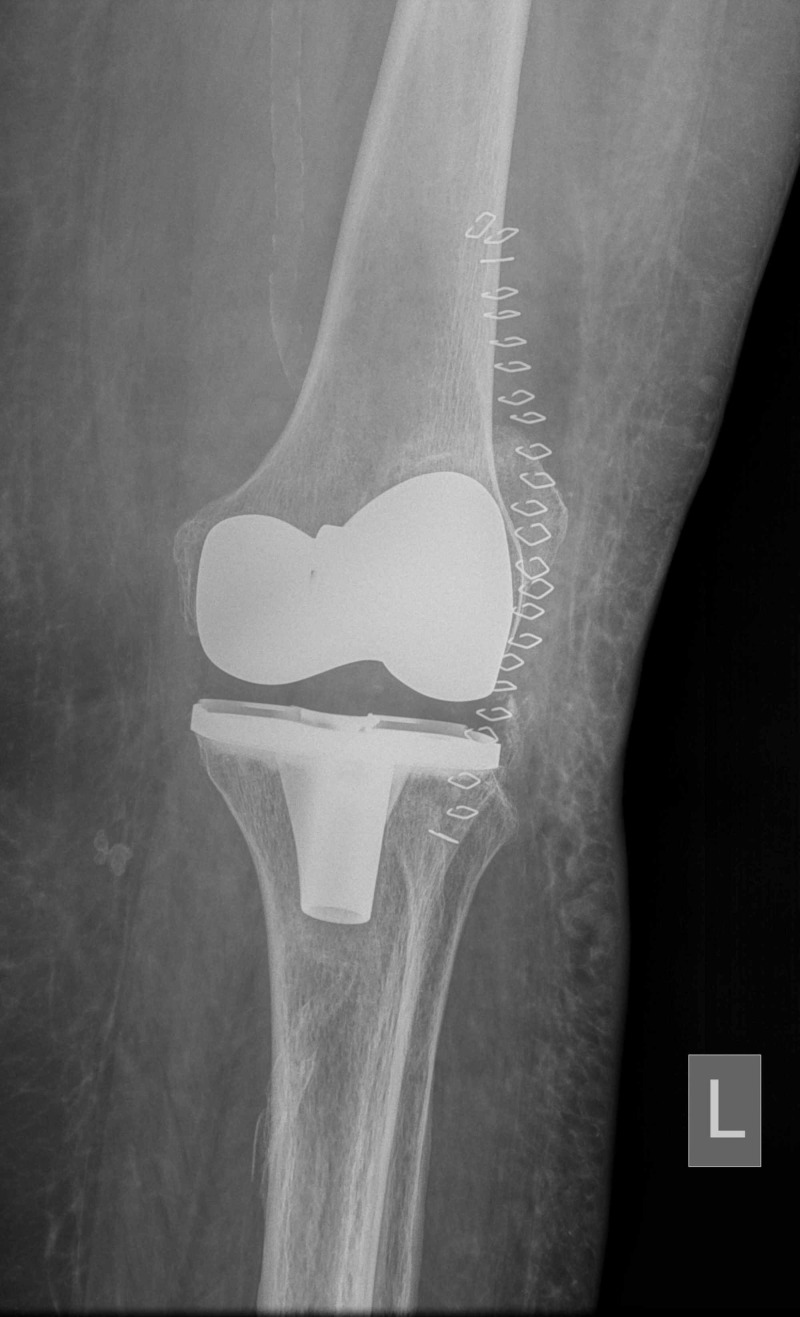
Immediate postoperative anteroposterior left knee radiograph L: left

**Figure 2:**
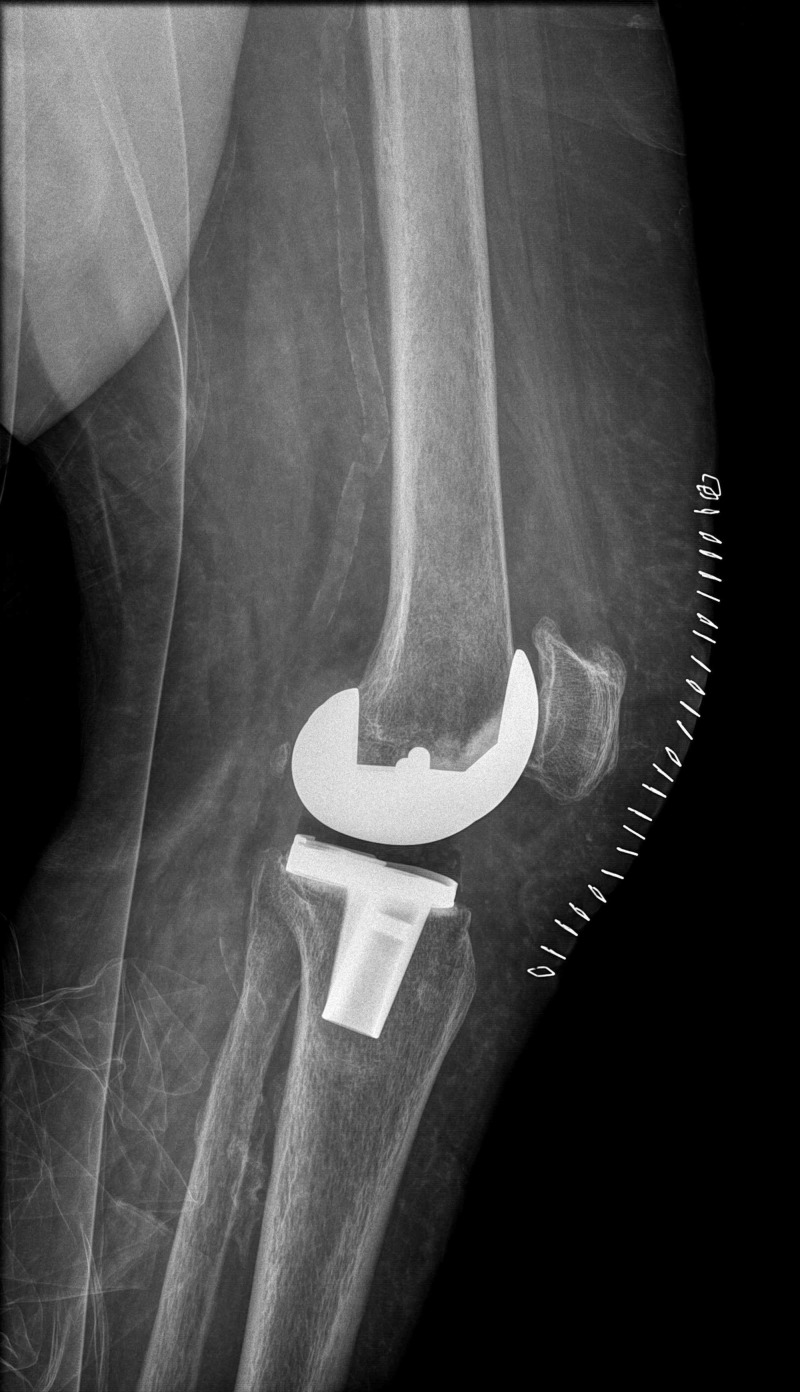
Immediate postoperative lateral left knee radiograph

On the tenth postoperative day, the patient turned up in our emergency department exhausted with high temperature up to 38.5°C, seeking orthopedic assistance for a knee infection. She was unable to ambulate, suffering from whole-body myalgia, generic malaise, and persistent cough but no dyspnea. On knee clinical examination, the wound was mildly inflamed with no effusion; thus, there was no suspicion of infection and aspiration deemed not necessary. The patient had an unrestricted passive knee range of motion 0-100 degrees, and the knee radiographs demonstrated stable, well-aligned implants with no signs of effusion (Figures 1-2).

Upon admission, blood tests revealed a white blood count of 7.400/mm^3^ (range:3.900-11.100/mm^3^) with a differential of 83% polymorphonuclears and 14% lymphocytes, reduced hemoglobin of 9.5 g/dl (range=11.8-14.8 g/dL) and hematocrit of 28.7% (range: 37-46%), normal platelet of 279 x 10^3^/mm^3^ (range 150-400 x 10^3^/mm^3^), and elevated serum creatinine 1.38 mg/dl (range: 0.57-1.11 mg/dl), creatine phosphokinase 299U/L (range: 29-168U/L), and lactate dehydrogenase 513U/L (range: 125-220U/L). The blood coagulation test and urinalysis were normal. The C-reactive protein test was not performed on admission.

**Figure 3:**
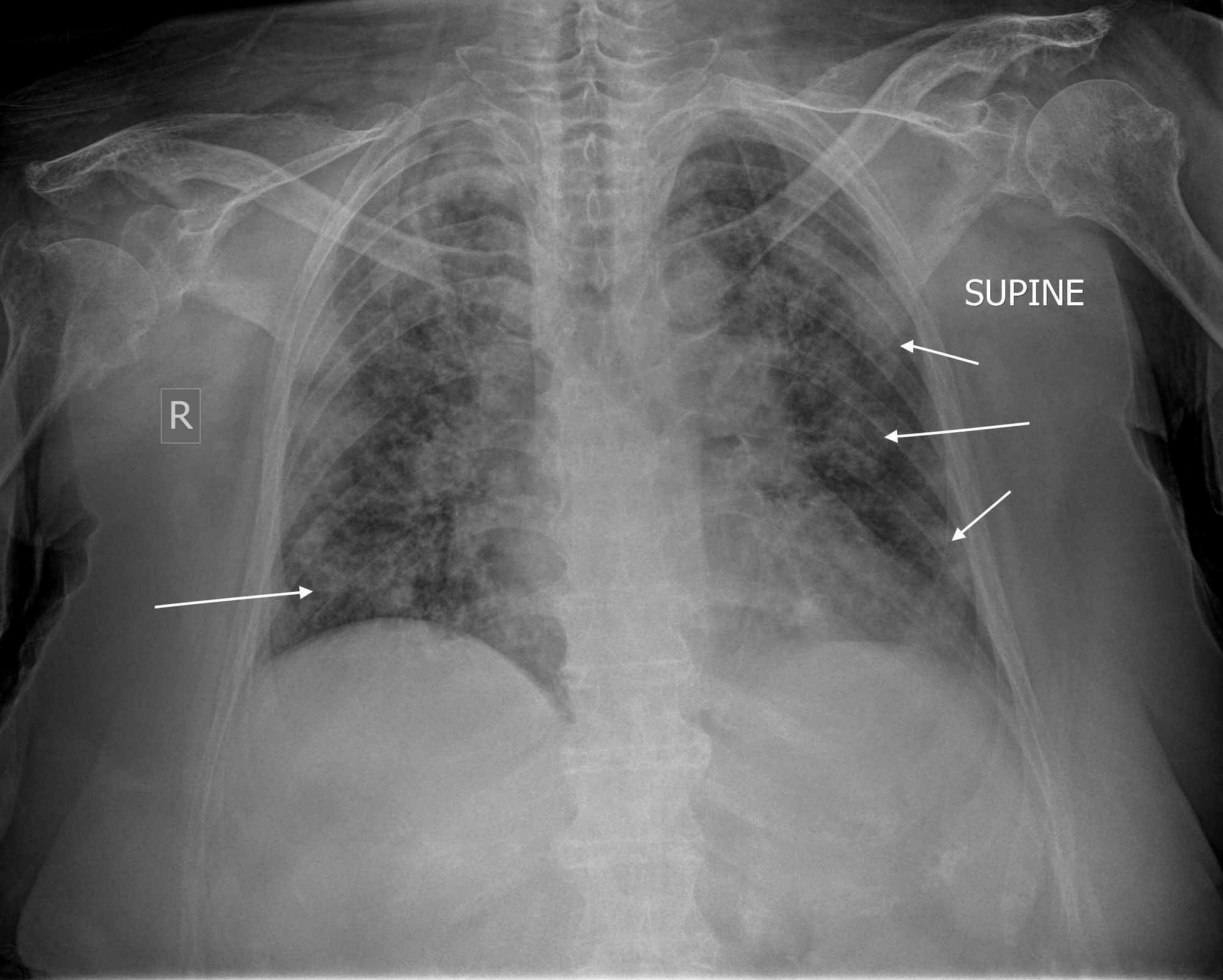
Anteroposterior chest radiograph demonstrating pneumonia of both lungs with typical ground-glass bilateral, multifocal, peripheral opacities (arrows) R: right

An infectious disease internist and pulmonologist were called upon to help diagnose and assist in managing the suspected infection. Chest radiograph revealed pneumonia of both lungs with ground-glass typical bilateral, multifocal, peripheral opacities due to an infectious organism (Figure 3). Consequently, coronavirus-19 disease (COVID-19) was suspected. The patient was admitted to a specially dedicated ward for suspected COVID-19 patients where pneumonia from SARS-CoV-2 was diagnosed. The patient was transferred to a COVID reference hospital and treated according to the COVID-19 protocol; she marginally escaped intubation though she went through significant respiratory distress for several days and is now slowly recovering.

## Discussion

Pneumonia is not an uncommon complication following orthopedic surgery [4-5]. The incidence of postoperative pneumonia following elective hip and knee arthroplasty was reported to be 0.34% [5]. Regardless of modern anesthetics and surgical progress, postoperative pneumonia caused by bacterial viruses and fungi is still related to significant morbidity and mortality [4-5]. Patients that develop postoperative pneumonia following elective lower limb arthroplasty surgery have a 19.4 times higher mortality rate [5]. Underlying causes, including chronic obstructive pulmonary disease (COPD), asthma, smoking, diabetes, obesity, heart and renal diseases, immunosuppression, and old age have been reported [4]. It is usually caused by bacteria; the majority of them being gram-negative aerobic, namely, Klebsiella, Pseudomonas, and Enterobacteria [4]. Viruses are atypical causative factors of postoperative pneumonia. To the best of our knowledge, this is the first reported case of postoperative pneumonia caused by the new SARS-CoV-2 masquerading as an acute postop periprosthetic joint infection following elective total knee arthroplasty.

The SARS-CoV-2 infection causes COVID-19, an unusual type of viral pneumonia that was first reported in China [6]. COVID-19 rapidly evolved into a worldwide health emergency leading, on 12 March 2020, the World Health Organization (WHO) to announce the COVID-19 outbreak a pandemic [7]. SARS-CoV-2 is highly contagious, spreads easily, sustainably, and efficiently, especially amongst susceptible people, increasing the risk of postoperative pneumonia for people undergoing elective surgery [8]. In the era of this pandemic, any elective surgery must be postponed, as the patient’s immunity is challenged by the trauma, leaving the body ill defended against a viral insult. The Ministry of Health of our country postponed all elective surgeries just one day following surgery of our patient.

In our patient, the age of 70 years was a moderate risk factor that is enhanced, however, by mild obesity and chronic insulin-dependent diabetes and, of course, the surgical trauma that increased the susceptibility to infection. The immune system of the patient was challenged postoperatively and became more susceptible to the highly contagious SARS-CoV-2.

The surgeon should always maintain the highest clinical awareness and level of suspicion, recognizing signs and symptoms of any infection. In our particular case, the absence of the typical clinical signs and symptoms of early postoperative joint infection led the surgeon to explore other reasons for the symptomatology. Malaise, myalgia, high fever, and coughing, along with proper auscultation, would have led to respiratory infection [8]. In addition, a low white cell count, with mild lymphocytosis, and a normal platelet count could help the differential diagnosis between bacterial and viral infections [8]. Besides, elevated creatine phosphokinase (CPK) and lactate dehydrogenase (LDH), along with the ground-glass, bilateral, multifocal, peripheral opacities in the chest radiograph recently correlated to the COVID-19 infection, could further help the diagnosis [8]. An early, proper surgical in-hospital assessment with possible knee aspiration would have established the diagnosis and is always advised in all arthroplasty patients regardless of social or medical restrictions. Asking patients to stay at home with fever following elective joint arthroplasty and offering antibiotics without a proper clinical examination and blood testing is ill-advised even in the era of social distancing measures due to the SARS-CoV-2 pandemic.

## Conclusions

COVID-19 symptoms can masquerade as an acute postoperative periprosthetic joint infection. In the era of the COVID-19 pandemic, a high index of suspicion for COVID-19 symptoms must be raised, especially in vulnerable patients, during the early postoperative period. Elective orthopedic surgery should be postponed, as the iatrogenic trauma challenges the immune system and the energy equilibrium may be shifted negatively, leaving the body ill-defended against a very aggressive insult of the sort of SARs-CoV-2. During the era of the SARS-CoV-2 pandemic, the social distancing rule “stay at home” applies to all except early postoperative patients with worsening malaise, myalgia, fever, and coughing, keeping in mind that COVID-19 may masquerade the symptoms of a postoperative periprosthetic joint infection.
